# Is self-rated health a valid measure to use in social inequities and health research? Evidence from the PAPFAM women’s data in six Arab countries

**DOI:** 10.1186/1475-9276-11-53

**Published:** 2012-09-17

**Authors:** Sawsan Abdulrahim, Khalil El Asmar

**Affiliations:** 1 Faculty of Health Sciences, American University of Beirut, Beirut, Lebanon

**Keywords:** Self-rated health, Social inequities in health, Arab region

## Abstract

**Introduction:**

Some evidence from high-income countries suggests that self-rated health (SRH) is not a consistent predictor of objective health across social groups, and that its use may lead to inaccurate estimates of the effects of inequities on health. Given increased interest in studying and monitoring social inequities in health worldwide, the aim of the present study was to evaluate the validity of SRH as a consistent measure of health across socioeconomic categories in six Arab countries.

**Methods:**

We employed the PAPFAM population-based survey data on women from Morocco, Algeria, Tunisia, Lebanon, Syria, and the Occupied Palestinian Territories (OPT). Multivariate logistic regression analyses were performed to assess the strength of the association between fair/poor SRH and objective health (reporting at least one chronic condition), adjusting for available socio-demographic and health-related variables. Analyses were then stratified by two socioeconomic indicators: education and household economic status.

**Results:**

The association between SRH and objective health is strong in Algeria, Tunisia, Lebanon, Syria, and OPT, but weak in Morocco. The strength of the association between reporting fair/poor health and objective health was not moderated by education or household economic status in any of the six countries.

**Conclusion:**

As the SRH-objective health association does not vary across social categories, the use of the measure in social inequities in health research is justified. These results should not preclude the need to carry out other validation studies using longitudinal data on men and women, or the need to advocate for improving the quality of morbidity and mortality data in the Arab region.

## Introduction

Self-rated health (SRH) is one of the most commonly utilized outcome measures in social epidemiology. It is often captured through a one-item question on survey instruments, in which participants are asked to rate their health in general on a scale from excellent to poor. Evidence abounds on the validity of SRH as a strong predictor of mortality, morbidity, and biological markers [1-3]. As such, the variable has been increasingly used in research to compare health between population groups and to monitor social inequities in health [4-7]. An assumption made in comparative studies that rely on SRH is that inter-group differences in subjective evaluations reflect unmeasured differences in objective health [8].

The universal validity of SRH as a strong predictor of morbidity and mortality, however, has recently come under question in a number of social epidemiological studies. As subjective health assessments are contingent upon social experience [9], researchers have argued that differences in SRH across social groups possibly represent systematic differences in meanings rather than differences in objective health. Initially, studies revealed a complex and often contradictory picture with respect to the association between SRH and mortality by gender, with some studies finding weaker associations for women compared to men while others showing the opposite [10]. Moreover, previous research indicated that SRH is a less valid predictor of objective health among immigrant and ethnic groups in the United States (U.S.) and Europe, and among indigenous populations in Australia [4,11-13]. In the U.S., non-English speaking immigrants tend to report lower SRH compared to their English-speaking counterparts [14-16], perhaps due to cultural and linguistic reasons [17]. As such, researchers have called for caution in interpreting the results of studies that use SRH in multicultural and multi-lingual contexts [13].

Evidence on the validity of SRH as an outcome measure in research designed to monitor health inequities across social groups is mixed. Early studies based on Swedish population data concluded that the predictive power of SRH for mortality did not differ by socioeconomic position (SEP) [18,19]. More recent evidence based on longitudinal population-based data from the U.S., Netherlands, and France, however, has shown that SEP does moderate the association between subjective and objective health, though the direction of the moderating effect depends on the SEP measure and the context [8,20-22]. For example, whereas the SRH-objective health association was weakest in the highest income category in France [21], it was strongest in the highest education category in the U.S. [22]. Further, a comparative analysis from a number of population-based data in the U.S. showed other inconsistencies in the predictive power of SRH across social groupings, suggesting that the variable is unsuitable for monitoring inequities over time [23].

These findings have substantive implications for future research on inequities. If the association between SRH and objective health differs across social groups, SRH would in some cases over-estimate and in other cases under-estimate the effect of inequities on health. As such, differences in SRH across SEP would be partly influenced by socially-determined threshold levels of what is considered good or poor health by members of these groups. For example, if the poor in any society have a higher threshold level for what they consider poor health, then studies on social inequities that rely on SRH can produce biased estimates. SRH may then capture both objective health differences due to socioeconomic inequities, as well as differences in subjective health assessments that are partly produced by these inequities.

Studies testing the validity of SRH have exclusively focused on high-income countries. With growing interest in examining and monitoring social inequities in health worldwide [24], however, investigating the validity of SRH in low- and middle-income countries where morbidity and mortality data are of poor quality or unavailable altogether is warranted. This is particularly the case in Arab countries of the Middle East and North Africa (MENA) region, where adult morbidity and mortality data are inadequate [25-27], and where researchers have increasingly turned to SRH data to examine differences in health between gender, national, and ethnic groups [28-30].

Whereas some recent evidence from the MENA region suggests that SRH is a valid measure of health for both men and women [31], to our knowledge, no study has yet investigated its validity as a consistent health outcome measure across socioeconomic groups in Arab countries. This investigation is timely and gains special importance in a region where the poor quality of existing morbidity and mortality data has hampered the examination of social inequities in health. The validation of other health outcome indicators, such as SRH, provides researchers a starting point from which to explore the available evidence and advocate for social policies to address health inequities. The aim of the present study was thus to evaluate the validity of SRH as a consistent health outcome measure across socioeconomic groups in six Arab countries-Morocco, Algeria, Tunisia, Lebanon, Syria, and the Occupied Palestinian Territories (OPT). Based on the best available population-based data in the region (collected by the Pan Arab Project for Family Health, PAPFAM), we assessed whether the association between SRH and physician-diagnosed chronic conditions among women was moderated by two measures of SEP (education and household economic status) in each of the six countries.

## Methods

### Study design

Data for this study were obtained from PAPFAM surveys conducted in Morocco (2004; N = 9869), Algeria (2002; N = 7399), Tunisia (2001; N = 3902), Lebanon (2004; N = 3499), Syria (2001; N = 6953), and the OPT which include the West Bank and Gaza (2006; N = 5098). For information on PAPFAM, please visit: http://www.papfam.org/pap/English/engindex.html. The cross-country surveys are population-based and employ a similar stratified, multi-stage, probability sampling design. In each country, data on family health were collected using four standardized instruments: a household questionnaire designed to gather household-level demographic and socio-economic characteristics; a questionnaire for ever-married women (15–54 years old), which contained questions on reproductive, maternal, and child health; an adolescent questionnaire; and an elderly questionnaire that focused on the health and quality of life of persons above the age of 60. PAPFAM did not collect data on adult men. In this paper, we drew on data from both the household and the women’s questionnaires.

"This study was based on secondary analysis of de-identified population data obtained from PAPFAM surveys conducted in Morocco (2004; N = 9869), Algeria (2002; N = 7399), Tunisia (2001; N = 3902), Lebanon (2004; N = 3499), Syria (2001; N = 6953), and the OPT which include the West Bank and Gaza (2006; N = 5098)."

### Measures

#### Subjective and objective health

SRH, the main outcome measure, was assessed by the following question: “In general, do you think your health is good, fair, or poor?” We dichotomized this variable into good versus fair/poor health. The main independent variable, objective health, was based on whether a respondent reported being diagnosed by a physician with at least one of five chronic conditions: diabetes, hypertension, asthma, cardiovascular disease, and arthritis.

#### Socioeconomic position

We included two main measures of SEP as effect modifiers. First, data on level of education were grouped into three hierarchical categories: primary or less, preparatory (a category that ranges from grade seven to grade ten), and secondary (high school graduation) or higher. We also included a measure of household economic status based on self-reported household income data in Lebanon (calculated in USD) and the wealth index in the five other countries, where direct income data were not available. For Morocco, Algeria, Tunisia, Syria, and the OPT, the wealth index was constructed using a list of 46 services and assets available in the household. Some of the services included were the availability of running water or electricity. Assets included whether a household had such items as a washing machine, refrigerator, or cell phone. Responses to services/assets were dichotomized into “available in the household” or “unavailable,” and the list of items was included in a principal components analysis. The index was then divided into three categories: poorest, middle, and richest. In Lebanon, total monthly income per household was categorized into poorest for households with income less than $433 per month (which is the legal minimum wage in Lebanon), middle for household income between $433 and $800, and richest for household income more than $800.

#### Covariates

We adjusted for four covariates in all multivariate analyses-age; two reproductive health measures (menopausal status and lost pregnancies); and smoking status. Age was included in all analyses as a continuous variable. Menopausal status and information on lost pregnancies were assessed using the following questions, respectively: “do you still have your period” and “did you have any pregnancy without a live birth.” Current smoking was assessed by asking whether a respondent currently smoked cigarettes, water pipe, or other tobacco. Current smoking and the two reproductive health measures were included in analyses as dichotomous variables (yes/no). Further, household ownership, work status, and region of residence were investigated. Household ownership and work status were included as dichotomous variables, yes/no. For Morocco, Algeria, Tunisia, and Syria, region of residence was categorized into rural versus urban. In the OPT, a third category, refugee camp, was added. For Lebanon, where data on rural versus urban residence were unavailable, region was grouped into four categories by governorate: Beirut and Mount Lebanon (most developed), Bekaa, North, and South.

### Statistical analysis

We carried out descriptive analyses for all the variables in the six country samples. Logistic regression models were then specified for each country to assess the strength of the association between fair/poor SRH and objective health, adjusting for age, the two SEP measures, and all other covariates. Following, to test whether the association between fair/poor health status and objective health was moderated by SEP, we specified a series of logistic regression models for each country stratified by education and household economic status. For example, to test whether the SRH-objective health association was moderated by education in Morocco, we ran adjusted logistic regression analyses separately for each education group in the country. The highest education and household economic status categories were set as reference. For validation purposes, we ran similar stratified logistic regression models testing the association between reporting poor SRH (instead of fair/poor) and objective health across education and household economic status categories in the six countries. All multivariate analyses were conducted on weighted data, taking into account unequal sampling probabilities. We considered a 95% confidence interval and a two-sided p-value of < 0.05 as indicative of statistically significant associations. Analyses were carried out using Intercooled Stata V.10.0.

## Results

The mean age for women ranged from 33 years in Syria and the OPT to 38 in Lebanon (Table 1). Reporting fair/poor health status was relatively high in all six countries. Higher proportions of women in the three North African countries (Morocco, Algeria, and Tunisia) reported fair/poor health (ranging from 53.9% to 71.7%), compared to those in the Eastern Mediterranean-Lebanon (42.3%), Syria (25.4%), and OPT (35.4%). This effect was mainly due to high levels of fair SRH; reporting poor SRH was low in all countries with the exception of Morocco (27.5%). Lebanon had the highest proportion of women reporting at least one chronic condition (15.8%) whereas Tunisia had the lowest (8.7%). With the exception of Lebanon (32.2%) and Syria (10.2%), rates of smoking among women in the four other countries were negligible (ranging from 0.4% in Algeria to 2.6% in OPT). The results show that Lebanon has the most favorable education profile for women (with 41.2% holding a secondary education or higher), followed by Tunisia (35.7%) and the OPT (32.5%). The proportion of women who completed a secondary or higher education was lowest in Syria (23.6%), while half of Syrian women received a primary education or less.

**Table 1:** Descriptive statistics of main variables of interest for the six countries

	**Morocco**		**Algeria**		**Tunisia**		**Lebanon**		**Syria**		**PT**	
	**(N = 9869)**		**(N = 7399)**		**(N = 3902)**		**(N = 3499)**		**(N = 6953)**		**(N = 5098)**	
**Age**	**34.64 ± 8.80**		**36.08 ± 7.79**		**36.46 ± 7.47**		**38.32 ± 8.58**		**33.07 ± 8.53**		**32.98 ± 8.27**	
	**N**	**%**	**N**	**%**	**N**	**%**	**N**	**%**	**N**	**%**	**N**	**%**
Self-rated health												
Good	2812	28	3346	44	1751	46	1965	58	5187	75	3295	65
Fair/Poor	7052	72	4041	56	2146	54	1397	42	1765	25	1803	35
Reporting at least one chronic condition	1387	14	847	12	331	8.7	524	16	626	9	609	12
Education												
Primary or less	1666	48	1603	39	1550	59	916	28	2730	53	1659	33
Preparatory	815	25	1365	34	125	5.6	945	30	1197	23	1766	35
Secondary or more	893	27	1172	28	748	36	1179	41	1215	24	1646	33
HH economic status												
Poor							1474	44				
Middle							1202	36				
Rich							669	20				
Working status (no)	8312	84	6739	91	3250	80	2773	82	5849	84	4548	90
Home ownership (no)	2612	28	1553	22	649	17	799	26	734	11	747	16
Region												
Urban	5053	58	4383	58	2380	67			3731	54	2709	55
Rural	4816	42	3016	42	1522	33			3222	46	1511	28
Refugee camp**											878	17
Region, Lebanon												
Beirut/Mt Lebanon							1423	41				
Bekaa							602	17				
North							724	21				
South							750	21				
Current smoker	74	0.7	31	0.4	65	1.7	1124	32	712	10	133	2.6
Ever lost a pregnancy (yes)	2305	24	1678	23	1126	29	1124	33	1423	21	2255	44
Menopausal (yes)			694	10	351	9	500	16	807	13	431	9.6

**: specific to OPT.

Table 2 presents results of fully-adjusted logistic regression models that show the association between reporting at least one physician-diagnosed chronic condition and fair/poor health. Reporting at least one chronic condition was positively and significantly associated with fair/poor health status in all countries. However, this association was weakest in Morocco (OR = 1.59; 95% C.I. = 1.22-2.06) and strongest in Lebanon (OR = 5.03; 95% C.I. = 3.76-6.75). Further, the results show that the associations between SEP and SRH are heterogeneous. The estimates were significant only in the three Eastern Mediterranean countries, but not in Morocco, Algeria, and Tunisia. In Lebanon, Syria, and OPT, the associations followed a gradient: with every decrease in the level of education, there was a graded increase in the odds of reporting fair/poor health. On the other hand, household economic status exhibited a gradient association with SRH in Morocco, Algeria, Tunisia, Syria, and OPT, such that the odds of reporting fair/poor health increased in a graded manner with decreasing household economic status. Lebanon is an exception, whereby the odds of reporting fair/poor health were not significantly higher for middle income women compared to the richest.

**Table 2:** Results of logistic regression models showing the association (OR and 95% CI) between reporting at least one chronic condition and fair/poor health

	**Morocco**			**Algeria**			**Tunisia**			**Lebanon**			**Syria**			**PT**		
	**OR**	**95%**	**OR**	**OR**	**95%**	**OR**	**OR**	**95%**	**OR**	**OR**	**95%**	**OR**	**OR**	**95%**	**OR**	**OR**	**95%**	**OR**
Reporting at least one chronic condition	1.59	1.22	2.06	4.66	3.33	6.52	4.17	2.79	6.23	5.03	3.76	6.75	4.79	3.80	6.04	3.76	3.04	4.65
Age	1.05	1.04	1.06	1.06	1.05	1.07	1.03	1.01	1.04	1.16	1.09	1.24	1.05	1.04	1.06	1.05	1.04	1.06
Education																		
Secondary or more																		
Preparatory	1.14	0.91	1.44	1.17	0.95	1.44	1.00	0.64	1.57	1.57	1.25	1.98	1.46	1.12	1.91	1.37	1.15	1.64
Primary or less	1.01	0.82	1.26	1.05	0.84	1.30	0.94	0.75	1.18	1.89	1.46	2.44	2.07	1.63	2.62	1.81	1.51	2.17
Working status																		
Working																		
Not working	0.98	0.79	1.22	1.18	0.92	1.53	0.87	0.68	1.11	1.19	0.91	1.55	1.18	0.93	1.51	1.35	1.06	1.72
HH economic status																		
Richest																		
Middle	1.25	1.01	1.56	1.26	1.04	1.52	1.27	1.02	1.57	1.32	0.98	1.78	1.40	1.15	1.70	1.53	1.28	1.83
Poorest	1.80	1.24	2.62	1.48	1.16	1.87	1.78	1.35	2.35	2.38	1.76	3.22	1.90	1.51	2.40	2.11	1.75	2.55
Region																		
Urban																		
Rural	0.94	0.71	1.25	1.20	0.99	1.46	1.15	0.91	1.44				0.78	0.66	0.94	1.01	0.86	1.19
Refugee camp**																1.26	1.04	1.53
Region, Lebanon																		
Beirut/Mt. Lebanon																		
Bekaa										0.74	0.55	1.00						
North										0.56	0.41	0.76						
South										0.46	0.33	0.63						
Current smoker	0.71	0.35	1.45	1.02	0.19	5.36	0.74	0.37	1.45	1.18	0.95	1.47	1.07	0.83	1.36	0.65	0.44	0.97
Ever lost a pregnancy (yes)	1.23	1.00	1.50	1.26	1.04	1.52	1.25	1.02	1.54	1.30	1.06	1.61	1.33	1.11	1.60	1.23	1.06	1.41
Menopausal (yes)				0.99	0.71	1.38	1.25	0.82	1.89	1.53	1.16	2.03	1.60	1.29	2.00	0.94	0.74	1.20

**: specific to OPT.

Other socio-demographic measures, such as working status (except in the OPT), home ownership, and region of residence were not predictive of SRH status. In the OPT, living in a refugee camp was significantly associated with higher odds of reporting poor health; whereas in Lebanon, living outside Beirut/Mount Lebanon was protective. Finally, being a current smoker was not associated with poor SRH in any of the six countries. In fact, current smoking was protective in the OPT.

**Figure 1:**
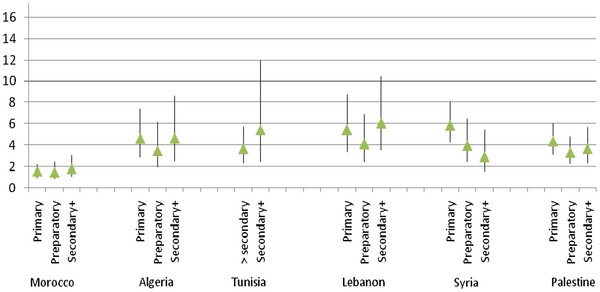
Multivariate associations (OR and 95% C.I) between SRH and objective health across educational categories for each country.

**Figure 2:**
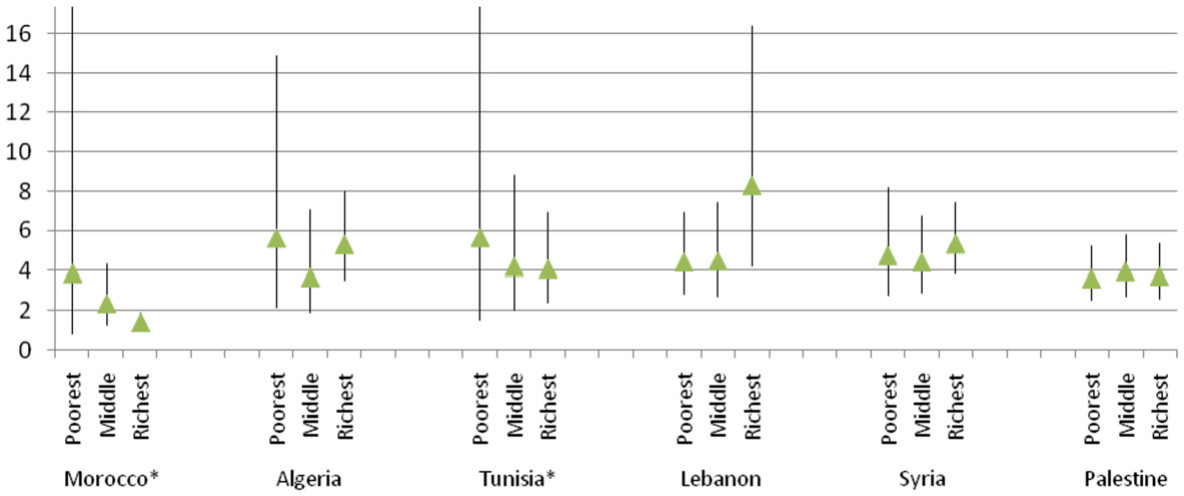
Multivariate associations (OR and 95% C.I) between SRH and objective health across household economic status categories for each country.

Figures 1 and 2 present results of logistic regression models fit to assess the strength of the association between fair/poor SRH and objective health (reporting at least one physician-diagnosed chronic condition) stratified by education and household economic status, respectively. An inspection of estimates and their 95% confidence intervals in Figure 1 reveals that the strength of the SRH-objective health association does not significantly differ across educational categories in the six countries. In Morocco, even though the association between SRH and objective health is weak, it is clearly of the same magnitude across the three educational categories. The association is both significant and of the same magnitude in the OPT. Though the estimates for the four other countries exhibit more variability - showing a U-shaped pattern in Algeria and Lebanon, a stronger association for the highest education category in Tunisia, and a stronger association for the lowest education category in Syria - the overlapping C.I.s signal the absence of significance. To confirm, we performed statistical tests for pairwise contrasts and found no significant differences between the estimates of the SRH-objective health association across educational categories, in any of the six countries. We also examined the results of logistic regression analyses predicting poor SRH (instead of fair/poor); with the exception of one category (secondary or higher education in Algeria), the association is not significantly different across educational categories in Morocco, Tunisia, Syria, Lebanon and OPT.

In testing the SRH-objective health association stratified by household economic status, the results show a similar pattern (Figure 2). In this case, the strength of the association between SRH and objective health is consistent in Syria and the OPT. The estimates reveal more variability in the four other countries, showing a stronger association in the richest household economic status category in Lebanon, a stronger association in the poorest category in both Morocco and Tunisia, and a U-shaped pattern in Algeria. Again, performing statistical tests for pairwise contrasts, we found no significant within-country differences between the estimates of the SRH-objective health association across household economic status categories.

## Discussion

Employing nationally representative, cross-sectional data gathered in six countries, we examined whether SRH is a valid measure to use in social inequities and health research in the Arab region. Our results show a strong association between fair/poor SRH and reporting at least one chronic health condition in Algeria, Tunisia, Lebanon, Syria, and the OPT, and a weak association in Morocco. The results further reveal no moderating effect by SEP on the association between SRH and objective health. In other words, SRH is not SEP-dependent but is a consistent measure of health across education and household economic status categories. These findings are in contrast to those reported in three studies published in 2007, which found differences in the predictive power of SRH for mortality [8,20,21]. They do, however, add some weight to two earlier studies showing that social inequities in subjective health status do reflect underlying inequities in objective health [18,19], and suggest that the use of SRH is justified in studies to document health inequities in Algeria, Tunisia, Lebanon, Syria, and the OPT. As interest grows in investigating inequities in a region that has been undergoing major social change, SRH data can provide important descriptive information on the impact of these inequities on health.

Before addressing study limitations and considering the implications of these results on future social inequities in health research, we discuss reasons for the high level of self-reported fair/poor health and other seemingly unexpected study findings. The high proportions of women in the PAPFAM surveys who selected fair or poor health is in line with findings reported in other studies in the Arab region [32-35], where, in some cases, levels of fair/poor SRH among women exceeded 50%. One reason may relate to the way in which SRH was measured. Compared to how SRH is customarily assessed in international studies, on a five-point Likert scale-poor, fair, good, very good, and excellent, the SRH question in the PAPFAM questionnaire offered three response options-good, fair, and poor. The use of a three-point scale may explain why a large proportion of women selected fair health status. Specifically, when respondents are offered three options, the middle category (fair) may be more frequently selected. This was the case in our study where levels of poor SRH were low in all countries (with the exception of Morocco) but levels of fair SRH were high. Similarly, in two studies carried out in Lebanon and Syria, the proportion of participants who selected the middle category on a three-point SRH scale were 31.9% and 35.6%, respectively [32,33].

As our study is based on secondary data analysis, it is not possible to conjecture as to why SRH was measured on a three-point and not a five-point scale in the PAPFAM surveys. This decision, however, may have been based on an understanding of cultural and gendered constructions of health in the Arab region. Indeed, in the pilot phase of a cross-cultural SRH study including Lebanon and Morocco, Parsons and Obermeyer noted that women in these two countries were reluctant to report very good or excellent health; consequently, the researchers used a three-point SRH scale [35].

In the present study, the use of a short scale did not affect the validity of SRH as a proxy measure of objective health in Algeria, Tunisia, Lebanon, Syria and, the OPT. In Morocco, however, the high level of poor SRH (27.5%) may explain the relatively weak association between this subjective measure and reporting at least one physician-diagnosed chronic health condition. This finding may be interpreted in one of two ways. The first is that poor health status may be over-reported among women in Morocco due to cultural factors or social conditions, and not actual levels of morbidity. The notion that poor SRH in some cultural contexts may reflect poor social wellbeing and not necessarily morbidity or future mortality risk has been addressed in studies among immigrants in the U.S. [14,36]. Evidence suggests that this may also be the case in the Arab region. In a four-country study where women were asked to evaluate their health status, Moroccan women were found to incorporate into their answers such factors as social isolation, family responsibilities, and poverty [35]. Despite the high level of fair/poor SRH in Morocco in our study, its association with objective health was not moderated by education or household economic status categories.

The second interpretation of our findings is that the high level of fair/poor health may reflect the true level of morbidity in Morocco. Given the objective health measure in the study is based on physician diagnosis, women may have under-reported chronic conditions due to lack of access to health care services. Evidence on access to reproductive health services in Morocco corroborates this interpretation, showing that a comparatively high proportion of women do not receive prenatal care during the course of their pregnancy [37-39]. Compared to Morocco, data from Tunisia, Lebanon, and the OPT show relatively higher levels of access to prenatal care and hospital deliveries [37,40]. As prenatal visits are occasions when chronic conditions can be diagnosed by a health care provider, the above-mentioned evidence explicates why the association between SRH and reporting at least one chronic condition was weak in Morocco but strong in the five other countries.

With the exception of the week (but consistent) SRH-objective health association in Morocco, the results of our study suggest that SRH is a good outcome measure to employ, at least to begin to describe the effects of social inequities on health in the Arab region. They, however, call for further research to better understand the strengths and limitations of this subjective health measure employing different types of data. The PAPFAM is a unique cross-national dataset which allowed us to examine the validity of SRH across social categories in a number of Arab countries. Still, the use of this data introduced several methodological limitations to our study. First and foremost, because the study sample is limited to women, it is not possible to make informed statements about whether the strength of the association between SRH and objective health would be the same for men. One study from Syria revealed that women were more likely to report poor SRH compared to men [32]. However, as the evidence on differences in the predictive power of SRH on mortality by gender is mixed [10], it is difficult to speculate whether the association between SRH and physician-diagnosed chronic conditions would be stronger or weaker among men in Arab countries, and whether this association would vary by education or household socioeconomic status.

Second, the cross-sectional nature of the PAPFAM data, whereby subjective and objective health were measured at the same point in time, may have caused an over-estimation of effects. We acknowledge that a rigorous validation of SRH as a robust proxy measure of morbidity or mortality across socioeconomic groups would best be based on longitudinal data. However, in light of the absence of population-based, longitudinal data in Arab countries, we argue that it is important to begin to explore social inequities in health in the Arab region even with the limited available data. Finally, a major limitation in our study is the use of another self-reported measure (physician-diagnosed chronic conditions) as a benchmark for SRH. The use of physician diagnosed conditions can introduce bias due to reporting heterogeneity or unequal health care access [41].

These limitations notwithstanding, the present study extends the small body of research that has investigated the robustness of SRH across social categories to the context of the Arab region. Our results suggest that SRH is a valid outcome measure to use in comparing the health between socioeconomic groups in five of the six countries under study. This opens the door for researchers who have long been interested in investigating the effects of social inequities on health in the Arab region but who have been constrained by the poor quality of morbidity and mortality data. SRH data can provide important preliminary evidence on the patterns and trends of health inequities in Arab countries, and ought to be used in informing social policies to alleviate these inequities. We emphasize, however, that given our conclusions are based on analysis of cross-sectional data, they should not preclude the need to carry out other SRH validation studies. Moreover, future research on this subject would benefit from qualitative inquiries seeking to disentangle the dimensions of subjective health assessments among different social groups. A few studies have examined cultural and religious meanings of SRH in some Arab countries [33,42], and among Arab immigrants in the U.S. [15,43]. Whatever the results of future longitudinal or qualitative studies may be, they should not be taken to question the value of SRH as a measure that captures important, but intractable, constructs of health and wellbeing [44].

## Competing interests

The authors declare that they have no competing interests.

## Authors’ contributions

SA conceptualized the study, supervised data analysis, and drafted the manuscript. KA performed the statistical analysis and contributed to drafting some sections of the manuscript. Both authors read and approved the final manuscript
